# Effect of Co-Administration of *Panax ginseng* and *Brassica oleracea* on Postmenopausal Osteoporosis in Ovariectomized Mice

**DOI:** 10.3390/nu12082415

**Published:** 2020-08-12

**Authors:** In Soon Kang, Taiwo Samuel Agidigbi, Young Min Kwon, Dong-Gyu Kim, Rang Ie Kim, Gyo In, Mi-Hyang Lee, Chaekyun Kim

**Affiliations:** 1 Laboratory for Leukocyte Signaling Research, Department of Pharmacology, Inha University School of Medicine, Incheon 22212, Korea; round001@hanmail.net (I.S.K.); samuelgeneration@naver.com (T.S.A.); soccer2509@naver.com (Y.M.K.); ehdrb3458@naver.com (D.-G.K.); good-angei@naver.com (R.I.K.); 2 Laboratory of Fundamental Research, Korea Ginseng Research Institute, Korea Ginseng Corporation, Daejeon 34128, Korea; 20109042@kgc.co.kr (G.I.); mhlee@kgc.co.kr (M.-H.L.)

**Keywords:** *Panax ginseng*, *Brassica oleracea*, postmenopausal osteoporosis, ovariectomy, bone mineral density

## Abstract

Postmenopausal osteoporosis is a common disorder resulting from increased osteoclastic activity. To determine the effect of *Panax ginseng* on postmenopausal osteoporosis, ovariectomized (OVX) mice were treated with 500 mg/kg/day *P. ginseng* extract (Pg) alone or in combination with hot water extract of *Brassica oleracea* (Bo) daily for 10 weeks, and the effect of the treatments on OVX-induced bone loss was examined. Bone weight, bone mineral density (BMD), osteoclast (OC) formation, OC marker expression, and biochemical parameters in blood were determined. OVX significantly increased body weight and decreased bone weight compared with those in the Sham group (*p* < 0.01). Pg or Bo alone did not affect OVX-induced bone loss, but a combination of Pg and Bo (Pg:Bo) recovered bone weight. The bones of OVX mice showed lower BMD than that of Sham mice, and the Pg:Bo = 3:1 restored the decreased BMD. Single treatment with Pg or Bo did not alter OC formation; however, the Pg:Bo = 3:1 inhibited OC formation. In addition, Pg and Bo lowered the OVX-induced elevation in blood glucose level. Thus, we suggest that Pg in combination with proper materials, such as Bo, might be a potential candidate treatment with minimal side effects protect against postmenopausal osteoporosis.

## 1. Introduction

Osteoporosis (OP) is a disorder characterized by low bone mass and structural deterioration of the bone microarchitecture, and it leads to an increased risk of bone fragility and fracture. Over 75 million people worldwide suffer from OP, of which 80% are postmenopausal women [1], and there is a direct relationship between the lack of estrogen and OP development [2]. In women, the two major causes of bone loss are menopause-induced estrogen deficiency and aging. Postmenopausal OP generally develops when, after menopause, estrogen levels drop precipitously. Senile OP is related to aging and it results from deficiencies in dietary calcium and vitamin D, and increased activity of the parathyroid glands. Calcium and vitamin D are implicated in gynecologic and reproductive conditions [3,4], and improve vasomotor disturbances, quality of life, and sexual function in menopausal women [5]. Moreover, high intakes of dietary vitamin D and calcium lowers risk of early menopause compared to low intakes [6].

*Panax ginseng* Meyer, which has been used intensively as a traditional herbal medicine for thousands of years [7,8,9], has become a most popular research subject in the field of natural medicine. The active components of *P. ginseng* include ginsenosides, polysaccharides, and flavonoids. In particular, approximately 40 types of ginsenosides have been identified and studied for their anticancer, antidiabetic, antioxidant, and immune regulatory effects [7,10,11]. The protective effect of ginseng on glucocorticoid-, inflammation-, radiation-, and ovariectomized (OVX)-induced OP has been reported [12,13,14,15,16,17,18]. *P. ginseng* prevented dexamethasone-induced apoptosis in osteoblastic MC3T3-E1 cells by lowering caspase-3 and -9 expression and increasing antiapoptotic genes, such as Bcl2 and Inhibitor of Apoptosis Protein [13]. Moreover, *P. ginseng* increased level and activity of alkaline phosphatase, osteocalcin, Runt-related transcription factor, and bone morphogenic proteins, thereby preventing dexamethasone-induced trabecular bone loss. Preventive effect of *P. ginseng* and *P. notoginseng* on bone loss has been extensively studied in OVX-induced OP model in rats [14,15,16,17,18]. *P. ginseng* decreased osteoclast (OC) numbers and OP in OVX rats [14], and saponins from *P. notoginseng* prevented OVX-induced decrease in bone mineral density (BMD) and deterioration in bone microarchitecture, and also suppressed OC turnover [15,17]. Moreover, ginsenoside Rg1 countervailed the decreased BMD in OVX rats [17], ginsenoside Rb2 revealed anti-osteoporosis effect by reducing oxidative damage and bone resorbing cytokines in osteoblast cells [19]. In contrary, ginsenoside Rb1 increased osteogenic differentiation and osteogenic proteins, however, did not halt osteoporotic bone loss in OVX rats [20]. However, despite accumulating data on the effect of *P. notoginseng* on OP in rats, the effect of *P. ginseng* on postmenopausal OP is not clearly defined; thus, we investigated the effect of *P. ginseng* on this disorder.

*Brassica oleracea* (cabbage) is highly nutritious and possess a wide range of bioactive compounds, such as glucosinolates, vitamins, phenols, anthocyanin, and carotenoids [21,22,23]. Approximately 200 types of glucosinolates have been identified in plants [24], and over 30 types are known to possess anti-cancer activities [25,26,27]. In addition, *B. oleracea* has anti-mutagenic, antioxidant, gastric and duodenal ulcer recovery, liver protection, and liver function improvement, and also inhibits microbial reproduction [28,29,30,31]. Although there are very few reports showing an anti-osteoporotic effect of *B. oleracea*, we found a paper showing that *B. oleracea* stabilize bone mass after menopause [32]. We also found protective effect on osteoporosis in rats in a preliminary study (data not shown); thus, we employed *B. oleracea* in this study.

Anti-osteoporotic drugs lead to improvements in OP, but they also cause many side effects, including gastrointestinal intolerability, osteonecrosis, osteosarcoma, and cancers [33,34,35,36]. Therefore, there is a great demand for safe and more efficacious anti-osteoporotic drugs. In this study, we evaluated the effect of *P. ginseng* as a safe medicine for long-term use in postmenopausal OP treatment using OVX-induced mice. Our results suggest that co-treatment of *P. ginseng* with *B. oleracea* restores OVX-induced decreases in bone weight and BMD and inhibits OC formation in vivo and in vitro, and thus can be used for treatment for postmenopausal OP.

## 2. Materials and Methods

### 2.1. P. ginseng and B. oleracea Extract Powders and Reagents

Standardized *P. ginseng* extract powder (Lot No. H1312-6010, ginsenosides: Rg1 + Rb1 + Rg3 > 7.0 mg/g) and *B. oleracea* extract powder were provided by Korea Ginseng Corporation (Daejeon, Korea). Dried *B. oleracea* was extracted twice with a 17-fold volume of distilled water for 8 h at 80 °C, filtered with 1-µm pore-size filter, and the filtrate was concentrated. The 17β-estradiol was purchased from Dalim Biotech (Preda^®^; Seoul, Korea) and heparin was obtained from Kimble Chase (Vineland, NJ, USA). All other chemicals were purchased from Sigma-Aldrich (St. Louis, MO, USA), unless otherwise stated.

### 2.2. OVX Operation in Mice

Seven-week-old female C57BL/6 mice were purchased from Orient Bio (Seongnam, Korea) and acclimated to the environment for 1 week prior to experiment initiation. The mice were maintained under pathogen-free conditions at a temperature of 23 ± 1 °C and humidity of 55 ± 5% under a 12-h light/dark cycle. The mice were provided a normal chow diet and water ad libitum during the experimental period. All procedures were conducted in accordance with the institutional guidelines approved by the Animal Care and Use Committee of Inha University (INHA 161214-465), and we followed the Animal Research: Reporting of In Vivo Experiments (ARRIVE) guidelines [37].

Sham and OVX operation were conducted on 8-week-old mice. The mice were operated under general anesthesia induced by intraperitoneal injection of 180 µL of ketamine–rompun cocktail containing 50 mg/kg ketamine (Yuhan, Seoul, Korea) and 20 mg/kg rompun (Bayer Korea, Ansan, Korea). The bilateral ovaries were exposed and removed from the mice, and the muscle wall and skin incisions were closed with 6-0 silk sutures (AILEE, Busan, Korea). In the sham-operated group, the ovaries were exposed using the same procedure, but were left intact.

### 2.3. Pg and Bo Treatment

A pilot study was conducted to determine the administration dose of Pg. OVX mice were treated with 100, 300, and 500 mg/kg Pg daily for 10 weeks. Although not significant, the mice treated with 500 mg/kg/day Pg showed higher recovery of OVX-induced bone loss compared those treated with 100 and 300 mg/kg/day Pg (data not shown). Thus, Pg and Bo were used at a concentration of 500 mg/kg/day in the subsequent experiments.

Mice were randomly assigned to experimental groups (10 mice in each group): Normal (naïve control group without incision), Sham (sham-operated control), OVX (OVX mice treated with vehicle), E_2_ (positive control treated with 1 mg/kg 17β-estradiol), Pg500 (500 mg/kg Pg), Bo500 (500 mg/kg Bo), Pg:Bo = 1:1 (250 mg/kg Pg + 250 mg/kg Bo), Pg:Bo = 1:3 (125 mg/kg Pg + 375 mg/kg Bo), and Pg:Bo = 3:1 (375 mg/kg Pg + 125 mg/kg Bo). E_2_, Pg, and Bo dissolved in water were administered orally at a volume of 0.2 mL/mouse once a day. From 1 week of operation, the mice were fed with the test substance daily for 10 weeks.

At the end of the administration period, the mice were anaesthetized by intraperitoneal injection of 180 µL of ketamine–rompun cocktail, and approximately 1 mL of blood samples were collected by cardiac puncture. The mice were sacrificed by cervical dislocation and bones were harvested. The animals were weighed weekly and administration dosages were adjusted according to weight.

### 2.4. Measurement of Bone Loss in OVX Mice

Animals were sacrificed 10 weeks following sample administration and femurs and tibias were dissected free of soft tissue. Bone weight and length were measured using a digital caliper (Mitutoyo, Kanagawa, Japan). OP is a disease in which the density and quality of bone are reduced, and density of bone is often evaluated by measuring BMD. Bone mineral content (BMC) and BMD of the right femur were analyzed using a Dual-energy X-ray absorptiometry (DXA, Norland Medical Systems, White Plains, GA, USA).

### 2.5. Histological Preparation and TRAP Staining

The femurs were fixed with 4% paraformaldehyde, decalcified in 10% ethylenediaminetetraacetic acid for 4 weeks, dehydrated in a graded series of 70–100% ethanol washes, and then embedded in paraffin. The paraffin blocks were cut with a microtome (SM 2010R; Leica, UK) to obtain 5-μm-thick tissue sections, which were then stained with hematoxylin and eosin (H&E). The sections were also stained with tartrate-resistant acid phosphatase (TRAP) by using a Leukocyte Acid Phosphatase kit according to the manufacturer’s instruction. Briefly, the sections were washed with phosphate-buffered saline (Hyclone, Logan, UT, USA), and fixed with a fixative solution containing citrate solution, acetone, and 37% formaldehyde. The fixed sections were subsequently incubated with TRAP staining solution for 60 min at 37 °C, and washed twice with water. TRAP-positive multinucleated cells with more than three nuclei were counted as OCs.

### 2.6. Blood Sampling and Analysis

After a 12-h fasting period, blood samples were collected by cardiac puncture in a heparinized tube, and plasma samples were obtained by centrifugation at 900× *g* for 5 min at room temperature. Next, plasma levels of alanine aminotransferase (ALT), aspartate aminotransferase (AST), glucose, and cholesterol were determined using Hitachi 7080 Automatic Analyzer (Tokyo, Japan).

### 2.7. OC Differentiation In Vitro

RAW 264.7 cells (ATCC, Rockville, MD, USA) were cultured in alpha-minimum essential medium (Hyclone) with 10% fetal bovine serum and 50 ng/mL receptor activator of nuclear factor kappa B (NF-κB) ligand (RANKL) for 4 days. The medium was replaced with fresh RANKL-containing differentiation medium at day 3. Cells were fixed and stained for TRAP, and formation of multinucleated functional OCs was assessed by counting the number of TRAP-positive cells containing more than three nuclei.

### 2.8. Statistical Analysis

All data were plotted as means ± standard error of mean (SEM), and group differences were determined by one-way analysis of variance (ANOVA) with Tukey’s multiple comparison test. Statistical significance was defined at *p* < 0.05.

## 3. Results

### 3.1. Effect of P. ginseng and B. oleracea on Body Weight

**Figure 1:**
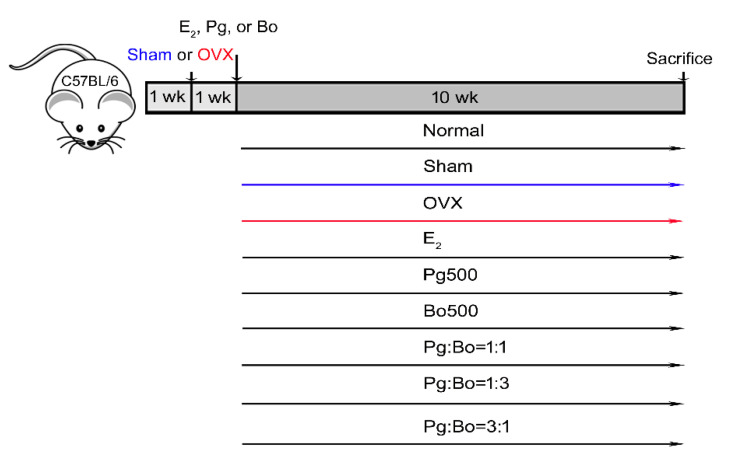
Treatment schedule for Pg and Bo in ovariectomized (OVX) mice. Seven-week-old female C57BL/6 mice were acclimated to the environment for 1 week and divided into 9 groups (*n* = 10/group). Sham and OVX operations were conducted and mice were treated with 500 mg/kg/day of Pg and Bo for 10 weeks from the 1 week of operation.

**Figure 2:**
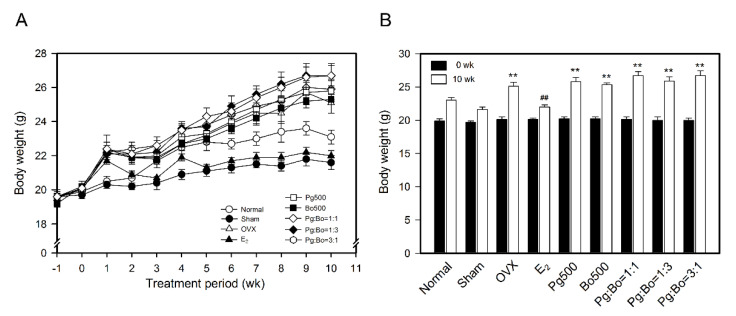
Effect of Pg and Bo on body weight of OVX mice. (**A**,**B**) Body weight was measured from the day of operation (−1 week) till the end of experiment (10 weeks) every week (*n* = 10). Normal, Sham, and OVX groups received water and sample treated groups received total 500 mg/kg/day of Pg, Bo, and Pg:Bo combination. All values are expressed as mean ± standard error of mean (SEM). ** *p* < 0.01 versus Sham, ^##^ *p* < 0.01 compared with OVX.

Menopause causes many physiological changes, in particular, increasing body weight, owing to low estrogen level. Mice were randomly assigned to experimental groups and were fed with the test substance daily for 10 weeks from 1 week of OVX operation (Figure 1), then the body weight was measured every week. The initial body weights of the mice showed no difference and body weight changes in all groups were proportional to time (Figure 2A). The body weight of OVX mice increased by approximately 16%, compared to that of the Sham group (OVX 25.1 ± 0.6 g vs. Sham 21.6 ± 0.4 g, *p* < 0.01). E_2_ treatment suppressed the OVX-induced increase in body weight to the level observed in the Sham group (E_2_ 22.0 ± 0.3 g, *p* < 0.01) (Figure 2). Mice in the OVX group were heavier than those in the Sham group, which were lighter than those in the Normal group. These results suggested that OVX surgery affected body weight and that estrogen deficiency caused weight gain. However, treatment with Pg500, Bo500, and Pg:Bo combination showed no effect on body weight; this result is contradictory to the reports that showed a significant recovery of body weight in OVX mice following treatment with *P. ginseng*.

### 3.2. Effect of P. ginseng and B. oleracea on Bone Weight and Bone Mineral Density

**Figure 3:**
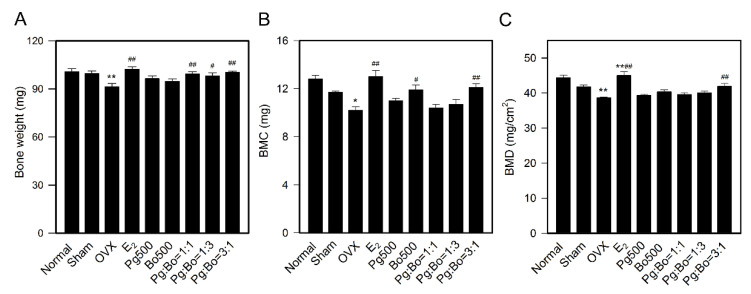
Effect of Pg and Bo on bone weight and bone mineral density. (**A**) Bone weight was measured on the day of sacrifice (*n* = 10). Bone mineral content (BMC) (**B**) and bone mineral density (BMD) (**C**) of right femurs were measured using a DXA (*n* = 8). All values are expressed as mean ± SEM. * *p* < 0.05 and ** *p* < 0.01 compared with Sham, ^#^ *p* < 0.05 and ^##^ *p* < 0.01 compared with OVX.

Osteoporosis is closely associated with low estrogen level. As expected, OVX mice showed significant reduction in bone weight compared with mice in the Sham group (OVX 91.4 ± 2.0 mg vs. Sham 99.7 ± 1.5 mg, *p* < 0.01), and treatment with E_2_ and Pg:Bo combination restored OVX-induced bone loss (Figure 3A), whereas single treatment with Pg or Bo did not show significant effect. In addition, there was no difference in bone length between the groups (data not shown).

BMD is used as an indirect indicator of OP. The BMD of OVX mice decreased compared to that of Sham mice (OVX 38.6 ± 0.2 mg/cm^2^ vs. Sham 41.8 ± 0.5 mg/cm^2^, *p* < 0.01) indicating a significant loss of bone mineral, and E_2_ restored OVX-induced bone mineral loss (E_2_ 45.0 ± 1.0 mg/cm^2^, *p* < 0.01 compared to OVX). Pg:Bo = 3:1 (108.5% of OVX) significantly recovered BMC and BMD (Figure 3B,C), similar to its effect on bone weight. The recovering effect of Pg:Bo combination on bone weight and mineral suggested that Pg in combination with Bo exerted protective effect against postmenopausal OP.

### 3.3. Effect of P. ginseng and B. oleracea on OC Formation

**Figure 4:**
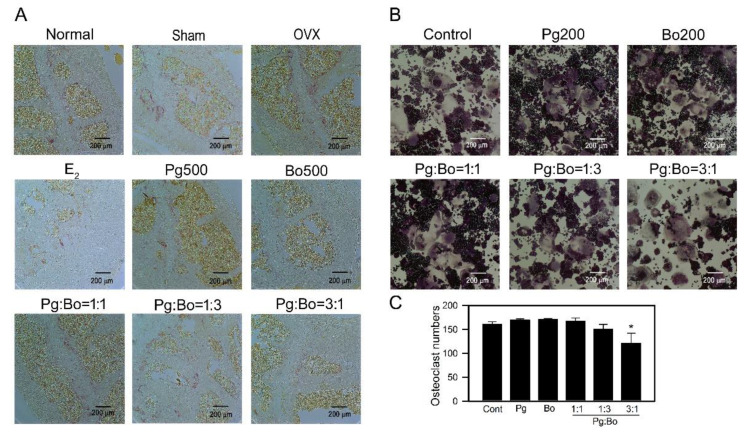
Effect of Pg and Bo on OC formation. (**A**) The femur sections were stained with tartrate-resistant acid phosphatase (TRAP) and histological analysis of each femur was performed under an Axioskop2 Plus microscope at 20× magnification. TRAP-positive cell numbers in selected femur fields were counted (*n* = 3). (**B**,**C**) RAW 264.7 cells were differentiated in the presence of 50 ng/mL receptor activator of nuclear factor kappa B ligand (RANKL) for 4 days and the differentiated OCs were stained with TRAP. TRAP-positive multinucleated OCs were counted (*n* = 3). All values are expressed as mean ± SEM, * *p* < 0.05 compared with control.

Bone sections were stained with H&E and TRAP. OCs were identified as TRAP-positive multinucleated cells located on the bone perimeter. OC localization was confirmed in a serial section stained for TRAP, and number of OCs was measured in a region of interest in five consecutive femur sections. The number of OCs increased in the femur of OVX mice, but E_2_ decreased OVX-induced increase in the number of OCs in vivo. In contrast, Pg and Bo did not decrease OVX-induced increase in the number of OCs (Figure 4A), but Pg:Bo = 3:1 combination decreased the number of OCs. In addition, OC formation from RAW 264.7 cells following RANKL treatment was determined in vitro. Cells were treated with Pg, Bo and Pg:Bo combination at concentrations of 50, 100, and 200 µg/mL, and the results showed that single treatments did not affect RANKL-induced OC formation. However, treatment with 200 µg/mL Pg:Bo = 3:1 combination significantly inhibited OC formation (Figure 4B,C), supporting the results of the in vivo study.

### 3.4. Effect of P. ginseng and B. oleracea on Blood Biochemical Parameters

**Figure 5:**
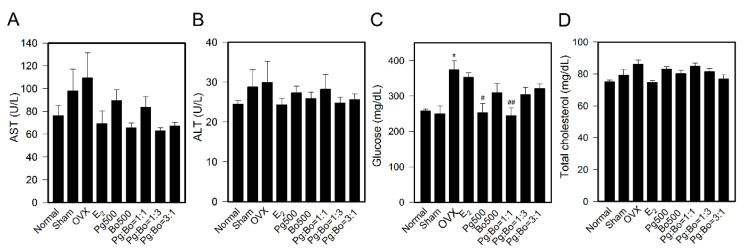
Effect of Pg and Bo on biochemical parameters in the OVX blood. (**A**), (**B**) Blood levels of liver enzymes aspartate aminotransferase (AST) and alanine aminotransferase (ALT), and glucose (**C**), and total cholesterol (**D**) were measured (*n* = 6~10). All values are expressed as mean ± SEM. * *p* < 0.05 compared with Sham, ^#^ *p* < 0.05 and ^##^ *p* < 0.01 compared with OVX.

We examined the association of menopause with biological factors, such as the blood levels of liver enzymes, cholesterol, and glucose. The Sham group, compared to the Normal group, showed increases in ALT (117.6%) and AST (125.3%) levels, but not in cholesterol (105.4%) and glucose (97.1%) levels, indicating organ damage. As shown in Figure 5, OVX mice showed significant increase in glucose (149.5%) level, but did not show changes in ALT, AST and cholesterol compared to Sham mice. Although the decrease was not significant, E_2_ decreased OVX-induced increases in ALT and AST levels, and Pg and Bo also decreased ALT and AST levels, with the Bo500, Pg:Bo = 1:3 and Pg:Bo = 3:1 treatments showing the most prominent effect (Figure 5A,B).

OVX, not Sham, increased glucose level and Pg500 significantly decreased OVX-induced increased glucose level almost to the level observed in Normal mice (Figure 5C). We assumed that the significantly increased glucose level in OVX mice was caused by estrogen deficiency; however, E_2_ did not lower OVX-induced glucose level. This indicated that the increased blood glucose level in OVX mice may not directly caused by decreased estrogen level, and that Pg may regulate blood glucose level in an estrogen-independent manner. In addition, OVX increased cholesterol level (114.6% compared to Normal), which was recovered by E_2_ and Pg:Bo = 3:1 to the level observed in Normal mice, although the recovery was not significant.

## 4. Discussion

At menopause, estrogen deficiency impairs normal bone remodeling cycle by increasing osteoclastic resorption activity without a corresponding increase in osteoblastic activity; therefore, the amount of bone resorbed is greater than the amount of bone deposited, leading to a net loss of bone [33]. OVX causes estrogen deficiency, and an OVX-induced osteoporotic mouse model has frequently been used. OP drugs and hormone replacement therapy lead to improvements, but they cause diverse unfavorable side-effects and need to be determined individual appropriate dose [33,34,35,36,38,39]; therefore, many menopausal women avoid taking these treatments for a prolonged period. Thus, we interested in the efficacy of herbal medicines and functional foods that are expected to have fewer adverse effects than chemical drugs and hormone replacement therapy.

In this study, we investigated the effect of *P. ginseng* and *B. oleracea* on postmenopausal OP, which was accompanied by decreased estrogen level. After OVX surgery, decreased estrogen level led to increased body weight, and Pg and Bo did not decrease the OVX-induced increase in body weight. This result is contradictory to reports showing the significant body weight restoring effect of *P. ginseng* in OVX mice [40,41]. *P. ginseng* restored the estrus cycle and estrogenic activity of OVX mice and inhibited OVX-induced obesity. Treatment with *P. ginseng* extract at 18.0 g/kg/day (36 times higher than Pg500) for 4 weeks showed the greatest decrease in OVX-induced increase in weight gain, whereas ginseng extract at 12.0 g/kg (24 times higher than Pg500) showed no significant effect [40]. Lee et al. [41] showed that treatment with 5% *P. ginseng* extract (50 g/kg, 100 times higher than Pg500) for 15 weeks resulted in 19% decrease in body weight, compared with that in OVX mice. From these results, we conclude that a certain amount of ginseng uptake is necessary to produce a noticeable effect against OVX-induced increase in weight. However, 36.0 g/kg is equivalent to 2160 g for a woman weighing 60 kg; thus, it is impossible to consume this amount of ginseng extract daily.

Bone mass and BMD are indicators of OP. Treatment with Pg:Bo combination restored OVX-induced bone loss (Figure 3A), and Pg:Bo = 3:1 recovered BMC and BMD to the levels observed in Sham mice (Figure 3B,C), indicating a synergistic effect of Pg and Bo in restoring BMC and BMD. There is much controversy on the effect of ginseng on BMD [15,18]. Treatment with 300 mg/kg/day *P. notoginseng* extract for 13 weeks restored decreased BMD in OVX rats [15], but treatment with 300~1000 mg/kg/day *P. ginseng* mixture for 7 weeks did not restore BMD in OVX rats [18].

Proper functioning of OCs, which are responsible for bone resorption, is necessary to maintain bone mass and BMD. The anti-osteoporotic activities of ginseng mixtures and different ginsenosides have been studied using cells and animals [12,42,43,44]. In this study, single treatment with Pg did not exert significant effect on OC formation; however, treatment with Pg:Bo = 3:1 inhibited OC formation in vivo and in vitro, suggesting that the administration dose of ginseng or its co-administration with a suitable material, in this case Bo, may be an important factor underlying the effect of ginseng on OC formation. Although we did not measure the effect of ginseng on osteoblast function and bone formation, the inhibitory effect of Pg:Bo combination on bone loss appeared to be somewhat related to bone production capacity. Further studies are required to clarify this relationship.

Estrogen deficiency alters glucose metabolism and body fat distribution [45]; at the postmenopausal period, body weight gain, insulin resistance, hyperlipidemia, and visceral fat accumulation occur. Furthermore, bone metabolism disorder in OP can influence global glucose homeostasis and energy metabolism [46]. *P. ginseng* has been traditionally used to treat diabetes, and ginsenosides are considered as major bioactive components mediating the antidiabetic effect of *P. ginseng* [47,48,49,50]. *P. ginseng* reduced the circulating levels of free fatty acids and triglycerides, and normalized hyperinsulinemia and hyperglycemia [41]. Consistent with these reports, Pg decreased blood glucose level to the levels observed in Sham and Normal mice. The antidiabetic actions of estrogen have been well recognized, and the incidence of diabetes rapidly increases at approximately the time of menopause [51]; however, in this study, E_2_ did not lower OVX-induced glucose increase. Although further investigation is required, it is possible that a dominant negative estrogen receptor β2 might be induced and involved in responses to E_2_ treatment [52]. Notable increases in ALT and AST levels are considered a sign of liver injury, but they can also indicate damage in another organs. AST and ALT levels increased at postmenopause [53], and have been reported to be related to insulin resistance [54]. Thus, we assumed that the increased levels of AST and ALT after operation contributed to the increased glucose level in OVX mice.

## 5. Conclusions

In this study, the most prominent effect of Pg on OVX-induced bone loss was shown by Pg:Bo = 3:1 combination. Pg:Bo combination protected OVX-induced bone loss by increasing BMD and inhibiting OC formation. Thus, we proposed that *P. ginseng* in combination with a suitable material, in this case Bo, might be beneficial in protecting against postmenopausal OP, and that finding the suitable material and determining the dose and ratio of the combination are critical in developing *P. ginseng* as an effective remedy for postmenopausal OP. However, the extracts of Pg and Bo contain various components whose effects are unknown, so it is not clear which substances are responsible for their action, and the characteristics of signaling mechanism by which these extracts inhibit OP remains to be determined. In addition, more studies, especially randomized controlled trials, are needed to apply the results of this study.

## Abbreviations

ALT	alanine aminotransferase
AST	aspartate aminotransferase
BMD	bone mineral density
Bo	*Brassica oleracea* extract
H&E	hematoxylin and eosin
OC	osteoclast
OP	osteoporosis
OVX	ovariectomized
Pg	*P. ginseng* extract
RANKL	receptor activator of nuclear factor kappa B (NF-κB) ligand
TRAP	tartrate-resistant acid phosphatase
